# Highly Incomplete Taxa Can Rescue Phylogenetic Analyses from the Negative Impacts of Limited Taxon Sampling

**DOI:** 10.1371/journal.pone.0042925

**Published:** 2012-08-10

**Authors:** John J. Wiens, Jonathan Tiu

**Affiliations:** Department of Ecology and Evolution, Stony Brook University, Stony Brook, New York, United States of America; Field Museum of Natural History, United States of America

## Abstract

**Background:**

Phylogenies are essential to many areas of biology, but phylogenetic methods may give incorrect estimates under some conditions. A potentially common scenario of this type is when few taxa are sampled and terminal branches for the sampled taxa are relatively long. However, the best solution in such cases (i.e., sampling more taxa versus more characters) has been highly controversial. A widespread assumption in this debate is that added taxa must be complete (no missing data) in order to save analyses from the negative impacts of limited taxon sampling. Here, we evaluate whether incomplete taxa can also rescue analyses under these conditions (empirically testing predictions from an earlier simulation study).

**Methodology/Principal Findings:**

We utilize DNA sequence data from 16 vertebrate species with well-established phylogenetic relationships. In each replicate, we randomly sample 4 species, estimate their phylogeny (using Bayesian, likelihood, and parsimony methods), and then evaluate whether adding in the remaining 12 species (which have 50, 75, or 90% of their data replaced with missing data cells) can improve phylogenetic accuracy relative to analyzing the 4 complete taxa alone. We find that in those cases where sampling few taxa yields an incorrect estimate, adding taxa with 50% or 75% missing data can frequently (>75% of relevant replicates) rescue Bayesian and likelihood analyses, recovering accurate phylogenies for the original 4 taxa. Even taxa with 90% missing data can sometimes be beneficial.

**Conclusions:**

We show that adding taxa that are highly incomplete can improve phylogenetic accuracy in cases where analyses are misled by limited taxon sampling. These surprising empirical results confirm those from simulations, and show that the benefits of adding taxa may be obtained with unexpectedly small amounts of data. These findings have important implications for the debate on sampling taxa versus characters, and for studies attempting to resolve difficult phylogenetic problems.

## Introduction

Biologists are becoming increasingly aware that accurate estimates of phylogeny are critical to many areas of research, from genomics to community ecology to the identification and spread of emerging pathogens. However, there are conditions where phylogenetic methods may give highly inaccurate estimates of phylogeny [1]. One such situation is when few taxa are sampled and branches among some of the sampled taxa are relatively long (i.e. many changes are expected or have occurred on these branches). Parsimony is thought to be particularly susceptible to this problem [2], but model-based methods (e.g. maximum likelihood, Bayesian analysis) may also fail to give accurate estimates under these conditions, especially when they utilize an incorrect model of evolution and when a relatively limited number of characters have been sampled (e.g. [3], [4]).

The problem of inaccurate estimation when branches are long can potentially be resolved by either adding more taxa to an analysis or by adding more characters. Adding taxa can potentially subdivide these long branches (e.g. [5]–[7]) effectively neutralizing the problem and rescuing the analysis (i.e. leading to accurate phylogeny estimation for the original set of taxa). However, some authors have argued that adding taxa can also decrease phylogenetic accuracy under some conditions [8], and that adding characters may be more helpful in some cases instead [9]–[10]. The question of whether it is more beneficial to add characters or taxa has proven to be one of the most contentious issues in systematics in recent decades (e.g. [5]–[18]). Yet, despite the potential relevance of taxon sampling versus character sampling to nearly all phylogenetic studies (especially those of higher taxa), the issue remains unresolved.

An important but typically unstated assumption in the debate over taxon sampling is that all added taxa must have complete data for all their characters in order to ameliorate the effects of limited taxon sampling. Importantly, nearly all assessments of the relative costs and benefits of adding taxa versus characters have been based on this assumption (e.g. [5]–[18]). But what if the added taxa needed to have only a fraction of the data present in the other taxa in order to subdivide long branches and rescue an analysis? For example, suppose the initial set of taxa had data from 4 genes, but some additional taxa were available with data for only one of these genes. If adding these taxa with partial data could still rescue the analysis, this would mean that the benefits of adding taxa could be achieved far more cheaply and easily than suggested by analyses in which all taxa are complete.

Despite its potential importance, the question of whether incomplete taxa can rescue molecular phylogenetic analyses from the effects of limited taxon sampling has been largely neglected in the recent literature (but for earlier discussions involving fossil taxa see [19]–[21]). One simulation study addressed this issue [4], and found that adding incomplete taxa could be surprisingly beneficial. For example, for simulated DNA sequence data, the benefits of adding taxa with only half their data present were often as high as adding complete taxa with all their data, and taxa that had 75% or even 90% missing data were often similarly beneficial. That study also showed that adding taxa that were only 10% complete was as or more beneficial than doubling the number of characters, even though only 30% as much added sequence data were required [4].

Although these simulations may have important implications for many phylogenetic studies, confirmation with empirical results is clearly needed. For example, the conditions simulated were not fully realistic (e.g. all added taxa had the same, relatively short branch lengths and were all evenly spaced along the long branches). Note that the question of whether adding incomplete taxa will improve estimation for the complete taxa is a separate question from that of whether incomplete taxa can be accurately placed in phylogenetic analyses (e.g. [22]–[31]) and whether adding characters with missing data improves accuracy (e.g. [30], [32]–[36]).

**Figure 1:**
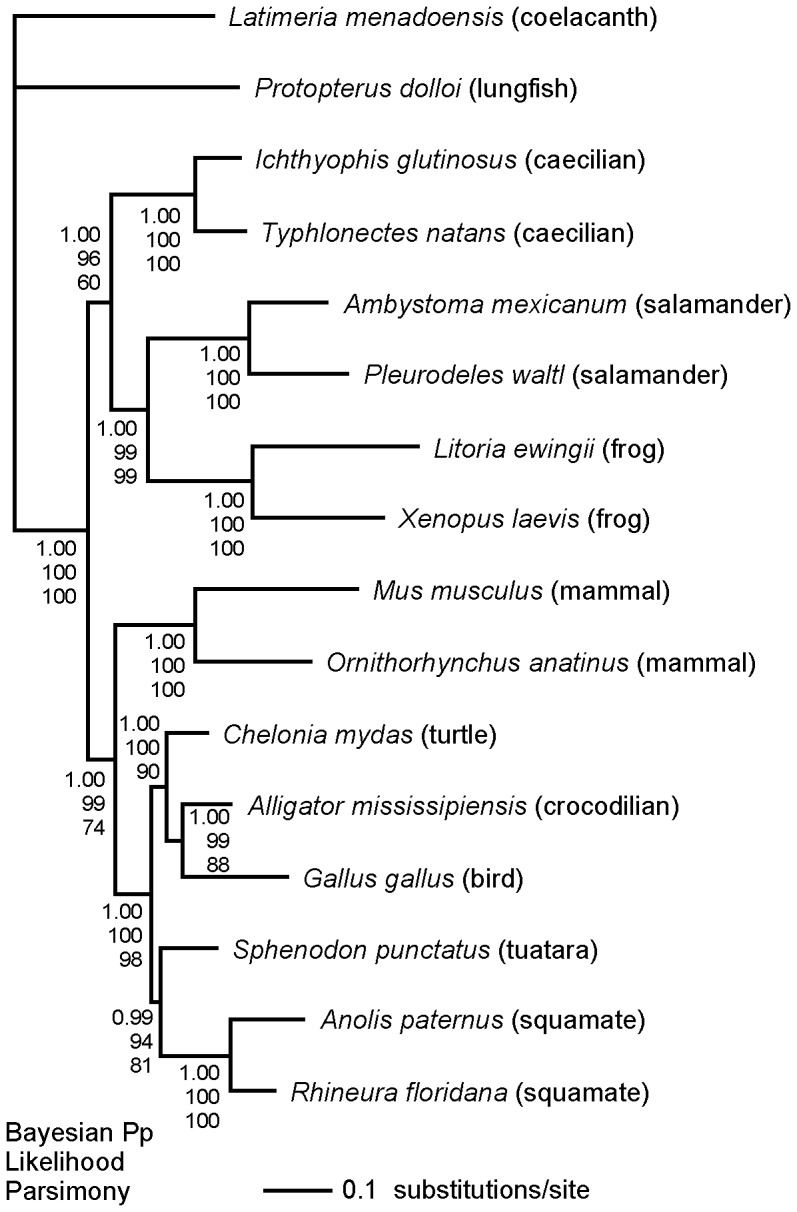
Phylogeny of 16 sampled vertebrates. Phylogeny of the 16 vertebrate taxa used in subsampling experiments. The same topology is estimated by Bayesian, likelihood, and parsimony methods, and the branch support from each method is shown (posterior probabilities for Bayesian analysis, bootstrap support for likelihood and parsimony). Branch lengths shown here were estimated using likelihood (absolute branch lengths from Bayesian analysis are somewhat longer, but relative branch lengths are effectively identical to those from likelihood).

In this study, we test whether incomplete taxa are able to rescue phylogenetic analyses from the effects of limited taxon sampling, using empirical molecular data from vertebrates. We take advantage of the fact that many aspects of higher-level vertebrate phylogeny are now relatively well established by both molecular and morphological data (e.g. [37], [38]). We subsample random sets of 4 taxa from a widely-used and well-sampled nuclear gene (RAG-1) from among a set of 16 taxa (Fig. 1) and find that for some sets of taxa, the estimated relationships are clearly incorrect. We then evaluate whether analyses including additional taxa (which have half or more of their character data removed and replaced with missing data cells), are able to recover the well-established relationships among the original 4 taxa in these cases (Fig. 1).

## Results

**Figure 2:**
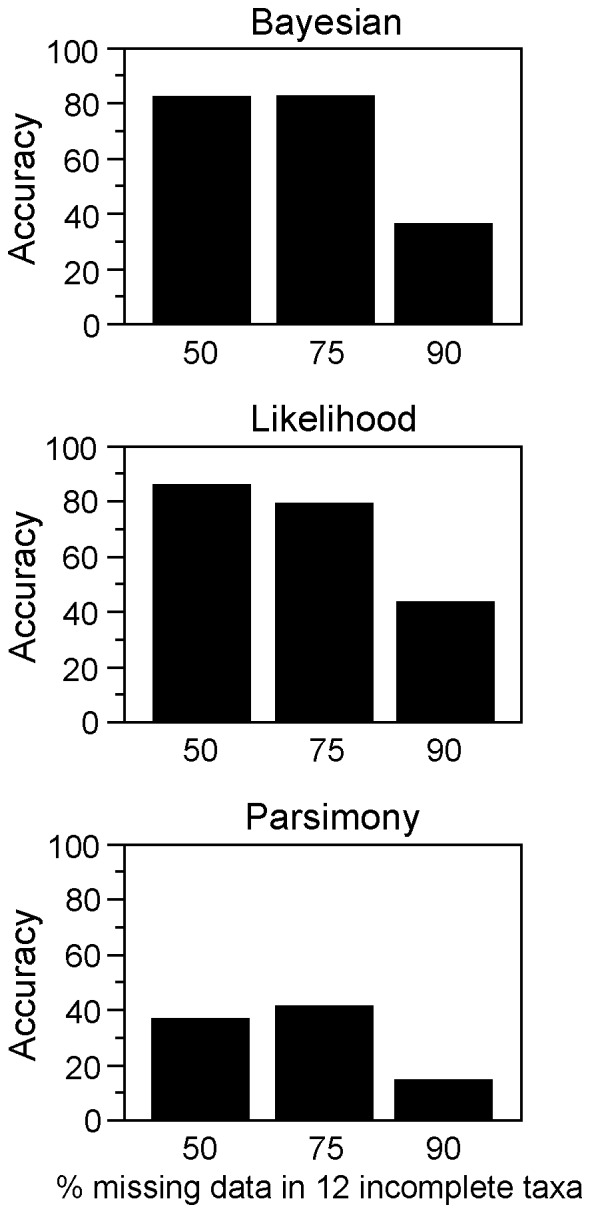
Major results of subsampling experiments. Major results of subsampling experiments from higher-level vertebrate phylogeny, showing that highly incomplete taxa can rescue analyses from the impacts of limited taxon sampling. Accuracy represents the proportion of replicates in which relationships among the 4 complete taxa are estimated correctly after adding 12 incomplete taxa, from among the set of replicates in which analysis of the 4 complete taxa alone yields an incorrect estimate. Thus, accuracy here represents the proportion of replicates in which the analysis of 4 complete taxa is initially incorrect but is “rescued” by addition of the 12 incomplete taxa (i.e. correct relationships among the original 4 taxa are restored).

The major results of the study are summarized in Figure 2. We initially generated and analyzed 100 random selections of 4 taxa, but we analyzed a total of 200 replicates with incomplete taxa by removing character data from either the 5′ or 3′ end of the gene in the incomplete taxa in each of the 100 original replicates (see Methods).

Bayesian analysis of the 4 taxa alone gives the incorrect phylogeny in 11 of 100 of the original 4-taxon replicates. The support for these incorrect relationships can be very high (range  = 0.47–0.99; mean  = 0.78). Addition of 12 incomplete taxa restores the correct relationships among the original 4 taxa in 82% of the 22 replicates when the 12 added taxa are 50% or 75% incomplete (Fig. 2). When the 12 added taxa are 90% incomplete, the correct relationships are restored in 36% of the 22 replicates (Fig. 2).

For likelihood, the analysis of 4 taxa gives an incorrect phylogeny in 7 of 100 replicates (all 7 are shared with those from the Bayesian analyses), sometimes with relatively strong support (bootstrap  = 28–88%; mean  = 53%). The analysis is often rescued by adding incomplete taxa (Fig. 2) when the added taxa are 50% incomplete (86% of 14 replicates), 75% incomplete (79%), and 90% incomplete (43%).

For parsimony, the analysis of 4 taxa alone gives an incorrect phylogeny in 12 of 100 replicates (mean bootstrap support  = 68%; range  = 52–90%). These incorrect replicates partially overlap those from Bayesian and likelihood analyses (7 of 12 and 4 of 12, respectively). Parsimony analysis is sometimes rescued by the added taxa (Fig. 2) when these taxa are 50% incomplete (38% of 24 replicates), 75% incomplete (41% of 22 replicates), and 90% incomplete (14% of 21 replicates). Overall, these empirical results confirm those from simulations [4], showing that Bayesian and likelihood analyses can potentially be rescued by adding taxa that have only 50%, 25%, or even 10% of their data present.

The results from adding complete taxa (Table 1) are generally similar to those from adding incomplete taxa (Bayesian analysis: 2 complete taxa rescue in 73% and 4 complete taxa rescue in 91% of 11 replicates; likelihood: 2 taxa rescue in 86% and 4 taxa in 100% of 7 replicates; parsimony: 2 taxa rescue in 75% and 4 taxa in 83% of 12 replicates). Comparing these results to those for adding 12 incomplete taxa shows that for Bayesian and likelihood analyses, a larger number of incomplete taxa can be as or more effective than a smaller number of complete taxa for rescuing analyses from the problematic effects of limited taxon sampling. Furthermore, we find that adding only 4 incomplete taxa gives similar results to those in which 12 incomplete taxa are added (Table 1). Thus, the benefits of adding incomplete taxa are not confined to cases when many incomplete taxa are added.

**Table 1:** The proportion of replicates in which analysis of the 4 complete taxa yields an incorrect phylogeny but addition of different numbers of complete or incomplete taxa leads to estimation of correct relationships among the original 4 complete taxa (i.e. the analysis is rescued).

	Phylogenetic method		
Sampling approach	Bayesian	Likelihood	Parsimony
12 incomplete taxa (50% missing each) added	82%	86%	38%
4 incomplete taxa (50% missing each) added	73%	79%	56%
2 complete taxa added	73%	86%	75%
4 complete taxa added	91%	100%	83%
Number of replicates	11	7	12

Conversely, our results show that adding incomplete taxa only rarely has an adverse impact on accuracy when the relationships among the original 4 taxa are correct (Table 2). Although the observation that adding these incomplete taxa sometimes decreases accuracy may be troubling, adding complete taxa also sometimes decreases accuracy for the original 4 taxa (at least for Bayesian and parsimony analysis). For Bayesian analysis, this occurs in 1% of 89 replicates when 2 complete taxa are added. For parsimony, this occurs in 1% of 86 replicates when 2 complete taxa are added and 1% of 88 replicates when 4 complete taxa are added.

**Table 2:** Percentage of replicates in which estimated relationships among the 4 taxa alone are initially correct, but adding incomplete taxa yields an incorrect estimate (for the 4 complete taxa).

	Phylogenetic method		
Sampling approach	Bayesian	Likelihood	Parsimony
12 incomplete taxa (50% missing each) added	0.6%	1.6%	0%
12 incomplete taxa (75% missing each) added	0.6%	1.0%	1.1%
12 incomplete taxa (90% missing each) added	1%	1.6%	2.3%
Number of relevant replicates	178	186	175, 175, 173

Overall, mean accuracy is increased for all 3 methods (for the 4 complete taxa) when the 12 incomplete taxa are added (Table 3). This increase is typically slight, because in the majority of replicates the estimated relationships among the 4 sampled taxa are accurate, and thus there is no potential for the analyses to be rescued and for accuracy to be increased. The increase for adding 2 or 4 complete taxa is typically higher than for adding incomplete taxa, but for Bayesian and likelihood analysis this difference is very small if the added taxa are 50% or 75% incomplete (for Bayesian analysis, accuracy is actually higher for adding 12 taxa with 50% or 75% missing data than for adding 2 complete taxa).

**Table 3:** Accuracy of phylogenetic methods for the 4 complete taxa (before and after addition of incomplete or complete taxa), including all 200 replicates.

	Phylogenetic method		
Sampling approach	Bayesian	Likelihood	Parsimony
4 complete taxa alone	0.89	0.93	0.88
12 incomplete taxa (50% missing each) added	0.98	0.98	0.93
12 incomplete taxa (75% missing each) added	0.98	0.98	0.92
12 incomplete taxa (90% missing each) added	0.92	0.94	0.89
2 complete taxa added	0.96	0.99	0.96
4 complete taxa added	0.99	1.00	0.97

Our results suggest that the inaccurate estimates obtained with limited taxon sampling may be caused by long-branch attraction (i.e. the tendency of phylogenetic methods to incorrectly place long branches together; see Methods). The mean branch lengths among the 4 sampled taxa are roughly twice as long as those for the 16 complete taxa (Bayesian: mean for 16 complete taxa  = 0.222: mean for 4 taxa  = 0.391: ML: mean for 16 complete taxa  = 0.122; mean for 4 taxa  = 0.238). Furthermore, in those replicates in which analysis of 4 taxa alone gives the incorrect phylogeny, the ratio of terminal to internal branch lengths is nearly twice as high relative to those cases in which the correct phylogeny is estimated (Bayesian: 2.80 vs. 6.80; unpaired t-test gives t-value: −6.828, d.f.  = 98; *P*<0.0001; likelihood: 2.80 vs. 6.77; t-value  = −5.793, d.f.  = 98; *P*<0.0001). This latter result suggests that long-branch attraction is the proximate source of error when few taxa are sampled (i.e. shorter internal branches and one or more longer terminal branches), and demonstrates that these errors are not merely random among replicates. We note that the mean estimated branch lengths in the trees with 12 highly incomplete taxa are similar to those in which all 16 taxa are complete, showing that adding incomplete taxa reduces branch lengths without grossly distorting them (Bayesian mean branch lengths for all 16 taxa, all taxa complete  = 0.222, 12 taxa 50% incomplete  = 0.216, 75% incomplete  = 0.221, 90% incomplete  = 0.203; likelihood, all taxa complete  = 0.122, 12 taxa 50% incomplete  = 0.123, 75% incomplete  = 0.135, 90% incomplete  = 0.136).

Some researchers may be concerned that adding many highly incomplete taxa will lead to phylogeny estimates that are generally inaccurate, even if the estimated relationships among the 4 complete taxa are improved. However, we find that the overall accuracy of the estimated trees can be relatively high (where accuracy is the proportion of nodes shared between the estimated phylogenies and the “known” phylogeny, averaged across all 200 replicates for each set of conditions and method), even when 75% of the taxa have 50% or 75% of their data missing (Table 4). There are notable decreases in accuracy when the 12 taxa have only 10% of their original data, however, as these matrices have less than a third of the original data present. Just considering the cases in which the estimate for the initial 4 taxa is incorrect (initial accuracy  = 0.00) gives similar results, showing dramatic increases in overall accuracy of the estimated trees when the 12 incomplete taxa are added (Table 4).

**Table 4:** Overall accuracy for estimated trees for all 16 taxa, when 12 of 16 taxa are incomplete, including all 200 replicates and including only the replicates in which the initial relationships of the 4 taxa are incorrect (accuracy  = 0).

	Phylogenetic method		
Sampling approach	Bayesian	Likelihood	Parsimony
All 200 replicates			
12 incomplete taxa (50% missing each)	0.93	0.94	0.88
12 incomplete taxa (75% missing each)	0.90	0.91	0.88
12 incomplete taxa (90% missing each)	0.70	0.65	0.50
Initial relationships incorrect			
12 incomplete taxa (50% missing each) added	0.92	0.91	0.86
12 incomplete taxa (75% missing each) added	0.90	0.92	0.84
12 incomplete taxa (90% missing each) added	0.70	0.60	0.48
Number of replicates	11	7	12

## Discussion

Our empirical results from analyses of higher-level vertebrate phylogeny show that highly incomplete taxa can be surprisingly effective at rescuing analyses from the misleading impacts of limited taxon sampling (i.e. they rescue an analysis by allowing recovery of the correct relationships among the original set of complete taxa). This pattern is most apparent for likelihood and Bayesian analyses, in which added taxa that each have 75% missing data rescue analyses in >75% of the replicates (Fig. 2). Importantly, we also found that adding incomplete taxa only rarely decreased accuracy when relationships among the complete taxa were correct (Table 2), and that overall accuracy increased relative to the accuracy for the 4 taxa when analyzed alone (Table 3). Thus, when the accuracy for the complete taxa was treated as unknown, adding incomplete taxa was (on average) either beneficial or harmless.

The overall accuracy of the estimated phylogenies for all 16 taxa does appear to decay substantially when most taxa have 90% missing data (especially for parsimony; Table 4). However, our results also suggest an obvious solution: simply prune the incomplete taxa from the estimated trees (Tables 1, 3). Thus, the benefits of these added taxa for subdividing long branches can be obtained, but the possibly incorrect placement of some of these taxa due to their limited sampling of characters need not impact the results. We note that here the taxa with 90% missing data have only 261 characters with data, and that the reduced accuracy of the 16-taxon trees with these taxa included (Table 4) may be explained by the limited resolving power of this very small number of characters, rather than by the missing data cells themselves being somehow actively misleading (see [22]). Clearly, if the missing data cells were themselves intrinsicallly misleading, they should be problematic when incomplete taxa have 75% missing data as well.

Overall, our results show that added taxa have the potential to be beneficial even when they are highly incomplete relative to other taxa (e.g. 50% or 75% missing data, but decreasing with 90% missing data). Thus, the benefits of taxon sampling may be obtained more cheaply and easily than considered in previous studies debating the pros and cons of adding taxa versus characters (e.g. [5]–[18]), which have implicitly assumed that added taxa must be complete to subdivide long branches. We do find that adding a limited number of complete taxa can be more beneficial than adding many incomplete taxa (Tables 1 and 3), but additional complete taxa may be unavailable in many empirical phylogenetic studies (otherwise they would have been included in the first place). Furthermore, we find that adding a limited number of incomplete taxa can also be helpful (Table 1), even if they are not as beneficial per taxon as those that are complete. Importantly, our point here is not that incomplete taxa are somehow better than complete taxa, but rather that adding incomplete taxa can be surprisingly beneficial despite their having much less character data than complete taxa.

Of course, no single empirical study can be generalized to all potential real-world scenarios, but the congruence between these empirical results and previous simulation results [4] makes a stronger case that both may be correct. Although our results here are based on data from only one clade of organisms (vertebrates), they confirm the results of a broader simulation study showing that incomplete taxa can rescue likelihood and Bayesian analyses from the misleading effects of limited taxon sampling under a diversity of simulated conditions [4]. The empirical results in our study suggest that those simulations adequately captured how these methods perform under these conditions in (at least some) real data sets, and show that a surprising result that was suggested to be possible in theory can indeed occur in the real world (Fig. 2). Further, both our results and those from the simulation study also agree that: (a) adding incomplete taxa does not consistently decrease accuracy for the 4 complete taxa for Bayesian, likelihood, or parsimony analyses, (b) potential benefits of adding taxa are often reduced when 90% or more of the data are missing in the incomplete taxa, (c) adding highly incomplete taxa is often more beneficial for likelihood and Bayesian analyses than for parsimony, and (d) the benefits of adding few complete taxa versus many incomplete taxa are generally similar [4] (although our results also show that the benefits of adding few complete taxa and few incomplete taxa can also be similar; Table 1). Admittedly, the present study and the simulations were matched in some aspects of their sampling design (i.e. both used 4 complete taxa and 12 incomplete taxa) in order to facilitate comparison. However, merely changing the number of taxa should not overturn the basic results.

Nevertheless, additional studies of this issue using simulated and empirical data sets would be desirable, especially to evaluate the effects of different genes (e.g. evolving at very different rates), different combinations of branch lengths (e.g. including species that are more closely related, rather than the relatively distantly related species included here), different placements of taxa along those branches, and different distributions of missing data (e.g. including missing data that occur in different characters in different taxa, as in many phylogenomic studies). Studies that specifically address the impact of adding incomplete taxa for the non-traditional datasets generated by RAD sequencing (e.g. [39]) would be particularly useful, as these datasets can have extensive missing data that are structured quite differently from those in the datasets considered here (e.g. [40]). Further investigation of the impacts of adding incomplete taxa in analyses of fossil data are also needed (e.g. [21]), and in analyses combining phylogenomic and fossil data (e.g. [4], [36]). Although we expect that the specific results will depend on the details of the data analyzed, our results from simulations and empirical data suggest that the same basic principles may apply widely (i.e. in those cases where adding taxa improves phylogenetic accuracy, adding incomplete taxa can potentially be beneficial, despite their missing data). For these future studies, we note that testing phylogenetic accuracy of different approaches using congruence with previous molecular and morphological results should offer a useful complement to simulation studies (e.g. [40]–[43]), and one that still remains underutilized.

Finally, a recent simulation study has suggested that missing data can be highly problematic for Bayesian and likelihood analyses [34], and that study has been used as justification for excluding taxa and characters with missing data in many empirical phylogenetic analyses (e.g. [44]). However, our results show that adding taxa with missing data can sometimes be beneficial (especially for Bayesian and likelihood analyses) and that excluding potentially useful taxa only because they have missing data cells might lead to less accurate estimates of phylogeny than could be obtained if these incomplete taxa were included. Thus, excluding taxa with missing data can actually have serious, negative consequences for phylogenetic accuracy. Clearly, incomplete taxa are not beneficial because they are incomplete (i.e. all other things being equal, it would be better to have more complete data and fewer missing data cells). Instead, our results show that, given the choice between adding incomplete taxa and adding no taxa at all, it may sometimes be better to add incomplete taxa.

## Methods

We began with the dataset of complete RAG-1 sequences compiled by Hugall et al. [38]. The RAG-1 alignment is relatively long (2613 characters) and is therefore comparable to a typical concatenated matrix of 3–5 gene fragments used in many empirical phylogenetic analyses.

From this original matrix we selected a set of 16 taxa (Fig. 1). This reduced number of taxa was intended to make the results more comparable to simulations of this same question [4]. In addition, the 16 taxa were chosen so that almost all relationships among the included taxa were uncontroversial, and could therefore be considered effectively known (e.g. monophyly of tetrapods, amphibians, frogs, salamanders, caecilians, amniotes, mammals, archosaurs, lepidosaurs, squamates). Less established aspects of vertebrate phylogeny (e.g. higher-level relationships within birds) were thus avoided. The only relationship that might be considered controversial among the 16 sampled species is the placement of turtles with birds and crocodilians, but this is now well supported by RAG-1 data [38] and by other analyses of molecular data (e.g. [37]).

Some of our selections of taxa were admittedly arbitrary, including the number of species in different groups (e.g. we sampled two species each of squamates, mammals, and the three major amphibian groups, but only one species each from turtles, birds, and crocodilians) and the specific choice of taxa within major groups. However, preliminary analyses of these data suggested that there were potential cases of long-branch attraction involving amphibians and mammals, and our sampling therefore emphasized these groups. We also selected the amphisbaenian squamate *Rhineura* (Florida worm lizard) specifically because previous molecular analyses suggested that it represented a problematic long branch [36]. Note that unless the analysis of the subsampled taxa alone gives misleading results, there is no potential for incomplete taxa to rescue (or fail to rescue) an analysis, and the data are not useful for our research question here.

Given the initial set of 16 taxa, we then performed subsampling experiments in which 100 unique sets of 4 randomly chosen taxa were analyzed. Four taxa is the smallest number of taxa in an informative unrooted phylogeny, and so use of 4 taxa should maximize the potential negative impacts of limited taxon sampling. We then analyzed each set of 4 taxa using Bayesian, likelihood, and parsimony methods (see details of methods below), and evaluated whether the estimate for the 4 taxa was correct, given the well-established relationships among all 16 taxa (note that analysis of the 16 taxa alone yields these same well-established relationships for all methods; Fig. 1). We then evaluated whether an analysis adding the other 12 taxa recovered the correct relationships among the original 4 taxa, after replacing some proportion of the character data for these 12 taxa with missing data cells. We tested the effects of adding taxa having 50%, 75%, and 90% missing data cells.

For each of the sets of 4 taxa, we evaluated the effects of removing data from the 5′ versus the 3′ end of the gene (and replacing them with “?”), thereby creating two replicates (or pseudoreplicates) for each of the 100 original data sets. Thus, for 50% missing data we deleted either characters 1–1306 (5′) or 1307–2613 (3′), for 75% we deleted characters 1–1960 (5′) or 654–2613 (3′) and for 90% missing data we deleted 1–2352 (5′) or 262–2613 (3′). We found that results differed somewhat depending on which set of characters had missing data (e.g. for Bayesian analysis with 50% and 75% missing data, 4-taxon analyses are always rescued when the missing data in the incomplete taxa are on the 3′ end, and those cases in which analyses are not rescued occur only when the missing data are on the 5′ end). Thus, the set of characters that are deleted can influence the results, and these paired replicates are therefore important.

All 12 incomplete taxa lacked data for the same set of characters within a given replicate (as done in most simulations [4]). In theory, missing data could also have been randomly distributed among cells in the matrix (although relevant simulations suggest that this would have relatively little impact; [4]). However, we consider the more realistic scenario to be the case when one fragment (or gene) is more broadly sequenced among taxa than others. We also note that lack of overlap in sampling of characters among taxa is a somewhat different issue than that of the amount of missing data per se. As is typical for empirical data sets, the complete data set included a small amount of missing data in each species, primarily associated with gaps (mean  = 1.1% missing data cells per taxon, range  = 0.2–3.6%).

Only a minority of the 100 original replicates yielded misleading phylogenetic results for the original 4 taxa (with the exact number depending on the phylogenetic method). Thus, although we evaluated accuracy for all 100 replicates after adding the incomplete taxa, our main results are based on those cases in which relationships among the original 4 taxa are incorrect. In theory, we could have analyzed more than 100 replicates overall or subsampled from more than 16 original taxa. However, such analyses would involve sampling different species from the same major clades (leading to similar combinations of branch lengths, regardless of species), and it seems very unlikely that such analyses would overturn our major results here.

For cases in which the estimate for the 4 taxa alone was correct, we also evaluated how often adding incomplete taxa led to an incorrect estimate for the original 4 taxa. If adding incomplete taxa frequently worsened the estimated relationships among the complete taxa when these relationships were correct, this disadvantage might outweigh the potential benefits of adding incomplete taxa in cases where limited taxon sampling leads to erroneous estimates.

We also evaluated the effects of adding either 2 or 4 randomly selected complete taxa to the original set of 4 taxa (note that adding all 12 taxa would simply give us the original tree; Fig. 1). This allowed us to compare the benefits of adding few complete taxa relative to adding many incomplete taxa. In addition, we tested the effects of adding only 4 incomplete taxa instead of adding 12 incomplete taxa. For these analyses we used taxa that were 50% complete and we selected the same 4 taxa that were used in the previous analyses adding 4 complete taxa (to allow for the most direct comparison). Finally, we tabulated the overall accuracy for each approach as the proportion of replicates in which the known “correct ” relationships for the original 4 taxa were estimated [4].

Each data matrix was analyzed using Bayesian, likelihood, and parsimony methods. For Bayesian analyses, we partitioned the data by codon position and applied the GTR + I + Γ model to each partition (with model parameters unlinked between partitions), following the model-fitting analyses of the full data set [38]. Bayesian analyses utilized MrBayes version 3.1.2 [45]. We performed two replicate analyses of 1 million generations each for each data set, sampling every 1,000 generations (but for analyses in which the incorrect tree was estimated, we confirmed that use of 2 million generations gave the same answer). Default values were used for all other settings and parameters. Stationarity was assessed based on plots of likelihood over time, and was confirmed using the average standard deviation of split frequencies between the paired analyses (<0.01) and examination of potential scale reduction factors. All analyses achieved stationarity, and we found that the number of generations used was more than adequate (especially given the relatively limited number of taxa and characters). Trees from the first 100,000 generations were typically discarded as burnin. The Bayesian tree was estimated from the majority-rule consensus of the post-burnin trees.

Maximum-likelihood analyses used RAxML version 7.03 [46]. The data were again partitioned by codon positions, and the GTR + Γ model was used (this model is recommended because the proportion of invariant sites, I, should be accounted for by 25 rate categories used for gamma, instead of the typical 4; [44]). For each data matrix we performed a heuristic search that combined 40 bootstrap replicates with 8 searches for the optimal tree (using the “-f a” option, although bootstrap values were not recorded). Again, these search parameters appeared to be sufficient given the limited number of taxa.

For parsimony analysis, we used PAUP* version 4.0b10 [47]. The shortest tree(s) were found by using 20 heuristic search replicates with tree-bisection-reconnection branch swapping and equal weighting among characters. In a few cases, parsimony analyses yielded multiple equally parsimonious trees, in which the consensus tree was unresolved for the relationships among the 4 complete taxa (e.g. only 1–3 replicates, in Table 2). These cases were not counted when assessing whether or not an analysis was rescued by the addition of incomplete taxa. We also estimated bootstrap support for those replicates in which the incorrect phylogeny was estimated for the 4 complete taxa, using 200 bootstrap replicates with branch-and-bound searching. We note that although we included parsimony for the sake of completeness and general interest, we do not necessarily recommend this method for molecular phylogenetic analyses of ancient groups, especially when there is the potential for long-branch attraction (given the well-known sensitivity of parsimony to this problem [2], [3]).

We also tested the hypothesis that long-branch attraction is the major cause of error when few taxa are sampled, and that the added, incomplete taxa rescue analyses by subdividing these long branches (e.g. [3]–[7]). Note that by “long-branch attraction” we simply mean the tendency of phylogenetic methods to erroneously place long, non-sister branches together under a given set of conditions (regardless of other issues, such as whether the methods would do so given infinite data or perfect fit between the model and data), following previous usage of the term (e.g. [4]). Our primary question here is simply whether incomplete taxa can potentially improve accuracy by subdividing long branches. For the 4-taxon case, long-branch attraction is expected most frequently when two or more terminal branches are long and the internal branch is short [2], [3]. We tested the hypothesis that the mean ratio of the terminal branch lengths (using the mean for the four terminal branches) to the internal branch length will be higher in those replicates in which the incorrect tree is estimated, using an unpaired t-test. However, we acknowledge that this analysis does not include the contribution of short, non-sister branches to problematic branch-length combinations. We also evaluated whether mean branch lengths (both internal and terminal) are longer in the 4-taxon data sets relative to those for the original 16-taxon data set, and whether adding the 12 incomplete taxa restored the mean branch lengths for all 16 taxa to values that are similar to those estimated for the 16 complete taxa. For these analyses, we focused on branch lengths estimated by Bayesian and likelihood analyses (given that parsimony ignores branch-length information). We note that the absolute branch lengths were somewhat longer in the Bayesian trees relative to the likelihood trees (e.g. mean  = 0.222 vs. 0.122 for complete data, all 16 taxa). However, the relative branch lengths are perfectly correlated (*r* = 1.000), and all of our analyses of branch lengths are based on separate comparisons for Bayesian and likelihood estimates.
